# G-Optrode Bio-Interfaces for Non-Invasive Optical Cell Stimulation: Design and Evaluation

**DOI:** 10.3390/bios12100808

**Published:** 2022-09-30

**Authors:** Vijai M. Moorthy, Parthasarathy Varatharajan, Joseph D. Rathnasami, Viranjay M. Srivastava

**Affiliations:** 1 Department of Electronic Engineering, Howard College, University of KwaZulu-Natal, Durban 4041, South Africa; 2 Department of Pharmacy, Annamalai University, Chidambaram 608 002, India; 3 Department of Electronics and Instrumentation Engineering, Annamalai University, Chidambaram 608 002, India

**Keywords:** electrical device characterization, neuronal cells, bio-interface, G-optrodes, non-invasive, optical stimulation, biocompatibility, microelectronics, nanotechnology

## Abstract

Biocompatibility and potential efficacy in biological applications rely on the bio-interactions of graphene nanoparticles with biological tissues. Analyzing and modulating cellular and device-level activity requires non-invasive electrical stimulation of cells. To address these needs, G-optrodes, bio-interfaces based on graphene, have been developed. These devices use light to stimulate cells without modifying their genetic code. Optoelectronic capabilities, in particular the capacity to transform light energy into electrical energy, will be maintained throughout the procedures of neural stimulation. G-optrodes have also been studied as thin films on a range of substrates, and they have been designed to function at a very small scale. This study examines the impact of G-optrode-based substrate designs on the optical stimulation of pheochromocytoma (PC-12). Graphene electrodes, known as G-optrodes, are responsible for converting light into electrical pulses with stimulating effects. G-optrode bio-interfaces provide a stimulus that is independent of wavelength range but is sensitive to changes in illuminance. The authors have performed a comprehensive investigation based on the correct effects of the medication in vitro, employing substrate-based G-optrode biointerfaces. In substrate-based systems, the authors have proven that graphene is biocompatible. PC-12 cells were cultured on graphene for 7 days. Based on the findings, 20-nm and 50-nm thick G-optrodes are being studied for possible use in biological and artificial retinal applications. The findings of this study highlight the significance of biocompatibility in the selection and use of G-optrodes for biomedical purposes.

## 1. Introduction

In recent years, various customized nanoparticles have been endlessly fabricated and researched for potential uses [1,2,3]. Brunton et al. [4] described electrodes for cortex stimulation that successfully and safely transmit energy to neural tissue. They have designed and tested the annular-shaped electrodes using Platinum Iridium (PtIr) that can be used in cortical visual prosthesis and they compared the device by utilizing porous Titanium Nitride (TiN). Their findings lend credence to the usage of PtIr annular electrodes with varying annular dimensions. Aryan et al. [5] demonstrated in their work that efficient high-density charge transfer to tissue is essential for brain stimulation. In those analyses, TiN material was used. According to their research, TiN electrodes can produce 0.2 mC/cm^2^ charge density in safe operation using square-shaped electrodes with 50 µm × 50 µm dimensions, which is less than other previously published studies. Their findings imply that rectangular electrode voltage outputs are preferable for maximum charge injection over a given lifespan. Platinum, iridium, and titanium nitride are common electrode materials, with the latter primarily utilized for in vitro monitoring purposes [4,5]. Green et al. [6] later discovered that Conjugated Polymer (CP) and implant environment characteristics impact surface lifetime. To highlight the future performance of such surfaces in neurostimulation systems. Furthermore, they compared the stability of Platinum (Pt) and Polyethylene Di-Oxythiophene (PEDOT) coatings in this work. According to the findings, PEDOT may be developed as a robust electrode layer that could be sterile and utilized safely and efficiently in neurostimulation devices.

Zhang et al. [7] examined graphene as a biocompatible surface coating for metal, which could be used in the biomedical field. According to in vitro cytotoxicity tests, graphene dramatically reduces Cu toxicity by impeding corrosion and lowering the concentration of Cu_21_ ions produced. At last, an animal experiment demonstrated that graphene effectively protects against Cu under in vivo environments. For recording applications, electrodes exhibiting low noise and impedance are preferred. Stimulation electrodes, on the other hand, must fulfill extra standards due to their active nature. It must be electrochemically reliable and robust in such a difficult condition for extended periods of time. To properly evoke nerve impulses, they must have both high-charge injections and energy storage space at the same time. Electrodes must also be compatible with the flexible properties of electrode substrates and able to survive long-term implantation. Electrical stimulation is suitable for various processes in cell physiology, notably stimulation, cell diagnostics, cell proliferation, ion homeostasis, protein expression regulation, and neural implants. Nowadays, organic electronics have begun to converge with biology and medicine, to build up a new generation of biosensors and to yield organic material-based devices for bio-interfaces. Later, Savchenko et al. [8] reported the optical stimulation of cells non-invasively using G-bio interfaces. In their first analysis, they demonstrated the optical stimulus of heart cells through the use of graphene-based interfaces in the substrate and soluble-based configurations. The effectiveness of stimulus through graphene-oriented bio-interfaces is self-reliant on wavelength range, but it could be altered by different illumination intensities. The ability to alter a cell’s viable state, and thus the overall function of an organ, by adjusting electrical signals via cell membrane potential. The technological methods used to achieve such monitoring must not jeopardize the integrity of the structure or the hereditary substance of the structure. This critical prerequisite presents a substantial problem. Later, Song et al. [9] described the creation of anti-HIF-1a anti-body-conjugated unimolecular polymer nanomicelles containing Paclitaxel for cancer-targeted treatment. These nanocomposites have effectively synthesized, bonded with stomach cancer cell lines MGC-803 particularly, incorporated, discharged PTX within tumor cells, and specifically terminated tumor cells according to the research results. Arrenberg [10] later demonstrated a genetically encoded, optically controlled pacemaker. In a preferential plane luminescence microscope, patterned emission is used. Their findings show that incorporating optogenetics and light-sheet microscopy could indeed expose how organ function emerges during growth.

According to Richardson et al. [11], infrared light can be used to directly stimulate neurons, whereas visible light could be used to stimulate neurons if the neural cells are genetically altered with a light-sensitive ion channel. Opto-genetics can accomplish highly precise stimulation with lower power, but only when combined with gene therapy-targeted implantation of a light-sensitive ion channel into the nervous system. For instance, certain electrical stimulation methods may have a negative impact on cell health and viability due to redox effects caused by Faradaic reactions or biological membrane rupture triggered by probing electrodes. Opto-genetics, or optical stimulus, is considerably more favorable to cells. Furthermore, an electro-optic stimulus needs large expression levels of foreign transmembrane ion-conducting proteins, implying that perhaps the researcher must modify a cell in order to influence its behavior [10,11,12,13]. Recent breakthroughs in nanotechnology have facilitated the development of integrated Nano-Particle Systems (NPS) on various substrates that have been shown to be a new generation platform for studying and controlling cellular function at the cell-device level. Researchers turned to graphene nanomaterials and their distinctive mix of electrical, mechanical, and optical capabilities to meet the challenge of designing a fully noninvasive stimulation approach [14]. Researchers developed novel processes and materials enabling precise diagnosis and avoidance of malignant illnesses, various medical devices, and artificial prostheses featuring high biocompatibility by integrating nanomaterials into the medical industry [15,16,17]. graphene and carbon nano tubes (CNT), for instance, are carbon-based materials that are well recognized for having extremely electrical, optical, and mechanical properties, as well as great light transmittance and electrical conductivity [18,19,20,21]. Therefore, in this regard, the biodistribution, size, and structure of carbon nanotubes impede their clinical applicability [22,23,24]. A scanning electrochemical microscope (SECM) has been adapted to perform the electroporation process of living yeast cells for the first time. The diameter of the area affected by the electrical pulse is about 25 times larger than that of the Au-ume ultramicroelectrode. Such a system could be promising for the selective treatment of selected cells in tissues and other sensitive biological systems [25].

**Figure 1:**
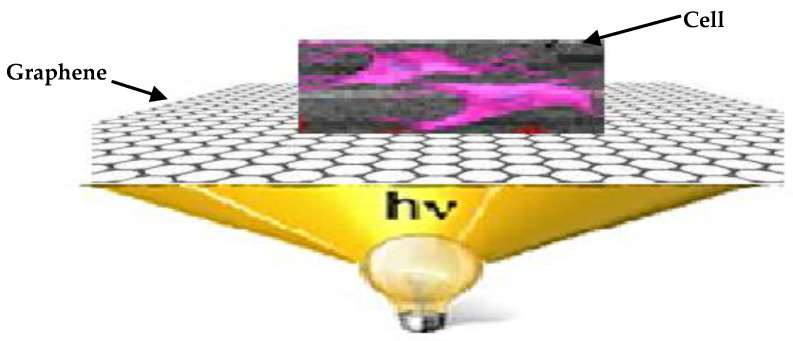
Schematic representation of the G-optrode driven optoelectronic device facilitating optic cell stimulation.

Graphene can rapidly transform light into electricity on a femtosecond time scale through the use of a hot-carrier multiplication process, making it particularly appealing for new applications in photonics and optoelectronics. Graphene sheets seem to be a subject of increasing interest, with substantial research being conducted for use in the development of novel composites [26,27]. These one-of-a-kind nanomaterials offer enormous promise in applications such as electrochemical sensors, power storage, catalysis, enzyme adsorption, bioimaging, biomedical applications, and biosensors [28,29,30,31]. Geim and Novoselov [32] extracted graphene from graphite utilizing mechanical cleavage and sticky tape for adhering graphene layer flakes. Graphene sheets and graphene quantum dots (GQDs) have remarkable qualities that make them more appealing for usage in the electrical, optoelectronic, and aerospace sectors. [33,34,35]. In this work, the authors developed an innovative solution to the problem of non-invasive optical stimulation of cells. A Graphene-based optical stimulator that is placed on the exterior of a physiologically and functionally viable cell is illustrated in Figure 1.

The biocompatibility of graphene-based nanomaterials in biological applications is a major concern. In established works of literature, the biological characterization of graphene-based materials is frequently contradicted. The effects of graphene nanoparticles on cell survival are dependent on a variety of parameters and experimental conditions [36,37,38].

Despite the basic potential for integrating graphene-based devices into living systems, few studies have been undertaken based on graphene for brain implant and cell diagnostic applications [39,40]. However, till now, no one has reported the optical stimulation of cells based on the thickness-dependent graphene bio-interfaces. For the first time, the authors describe the impact of graphene on the thickness-dependent configuration of PC-12 cells. This will work as a preventative measure in regulating active cellular functions. In this present research work, G-optrodes in a substrate base have been proposed as an aspirant for direct interfacing with a neuronal system. The interaction between the graphene electrode and neural cell lines or living tissue entails the optoelectronic properties of graphene remaining intact during cell culture and in the biological environment. The effects of in vitro interactions between graphene-based nanomaterials and cells are examined in this work.

Furthermore, the G-optrodes have high electrical conductivity, making them a great choice for stimulating the cells optically, for tissue engineering, and bio-sensing purposes. These discoveries might provide opportunities for the sustainable development of graphene-based nanomaterial structures and biological interfaces for biomedical applications such as retinal implants. This paper is structured as follows: Section 2 focuses on the materials and procedures used in this work, as well as sample preparation for experimental studies. Section 3 realizes the electrical characterization of the G-optrode thin film and evaluates the bioorganic interface behavior of G-optrodes. Section 4 analyses and evaluates the bio-interface behavior of G-optrode films via optical stimulation using Pc-12 cells. Finally, Section 5 concludes the work and recommends the future aspects.

## 2. Materials and Methods for G-Optrode Electrical Characterization and Cell/Graphene Interfaces Preparation (Sample Preparation)

This research focus of this research work is on analyzing and realizing electrochemical stability and biocompatibility of a G-optrode layer over a glass substrate. The materials employed in this work are of analytical grade. In authors’ previous work [41], they used these materials and procedures in the preparation for biocompatibility studies, which have been outlined in brief.

### 2.1. G-Optrode Thin Film Physical Realization

**Figure 2:**
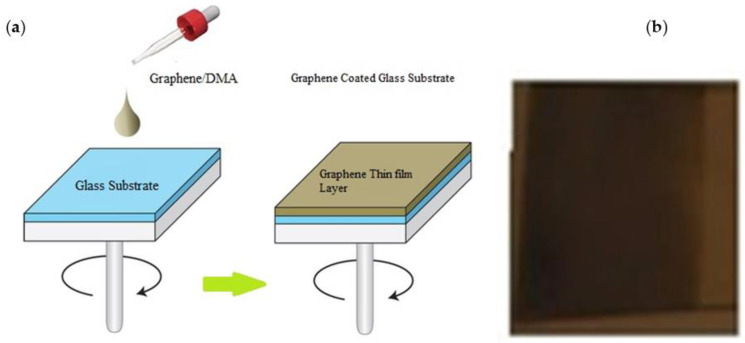
(**a**) Schematic representation for thin film fabrication and (**b**) fabricated sample image.

The materials used in this work were utilized in accordance with the manufacturer’s instructions. Glass substrates were used to prepare the samples. By spin coating graphene sheets on a glass substrate, the researchers developed substrate-based G-optrodes for bio-interfaces. Before cell culture, the prepared samples were thoroughly cleaned using ultrasonic baths, isopropanol, and acetone rinses to eliminate any unavoidable residues. To begin, the graphene was dispersed in a solvent, Di-Methyl Acetamyde (DMA), to provide a uniform coating and adherence across the substrate. Initially, the researchers mixed the graphene powder (56.5 mg) in 20 mL of DMA for 2 h with a probe-type sonicator [42]. DMA had been used to disperse graphene, and thus the mixture was deposited over a glass substrate using the spin coating technique for 30 s at various spin rates ranging from 1000 to 1600 rpm. After spin coating, researchers used hot air ovens to dry the graphene sheets for 10 min at 80 °C. Following the deposition of graphene on glass substrates, the annealing procedure is carried out in a 400 °C hot air oven. Thermal annealing was performed on the prepared samples in a 25 °C oven for 20 min. The annealing process served several purposes, including sterilization, improved film morphology, improved charge photo generation efficacy [12], and equipping the samples for further cell growth by eliminating any residues of chemical reagents including acetone, methyl alcohol, chlorobenzene, and other highly toxic organic solvents, as well as sterilizing the substrate. The coated graphene exhibits enhanced homogeneity and adherence to the glass substrate after the post-annealing procedure. Figure 2 illustrate a schematic depiction of the film fabrication process as well as an optical view of the film over a glass substrate, respectively. Annealing increases the conductivity of a film while decreasing its sheet resistance, whether it is pure graphene or graphene that has been chemically altered [reduced graphene oxide (rGO)]. At various spin speeds, a spin-coated graphene film is evaluated for sheet resistance and optical transmittance. G-optrodes with thicknesses of 20 nm and 50 nm were created by spin coating them at 1000 rpm and 1600 rpm, respectively.

### 2.2. This Present Research Work Analyses the Effect of G-Optrode Thin Film

The effective organic bio-interface system should fulfill the following requirements: the device must be unaffected or free of defects by sterilization methodologies; the graphene film/G-optrode must retain its optical characteristics and morphological properties until being immersed in Phosphate-buffered saline (PBS); cell proliferation and percentage of viable cells should be pretty much formed throughout the thin film layer, and adequate functional requirements of such transparent electrode must be demonstrated.

**Figure 3:**
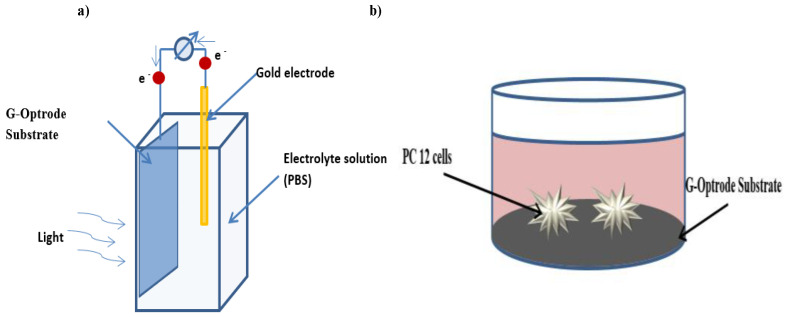
G-optrode bio-interface experimentation setup (**a**) solid-liquid devices used in this work are depicted schematically. (**b**) Methodology used in the work is depicted schematically.

The authors have realized G-optrode samples fabricated with thicknesses of 20 nm and 50 nm without the neuronal interface to validate the first two cases. As illustrated in Figure 3a, the researchers utilized hybrid bio-interface devices kept in PBS solution as an electrolytic cathode. Because it is a crucial ionic component in numerous extracellular fluids, PBS was employed to comprehend the principle of hybrid optoelectronic functioning. The authors began by submerging the G-optrode bio-interfaces in a PBS solution for 30 days to ensure their stability.

The graphene layer coating over the glass was found to be stable for both the 20 nm and 50 nm samples. Figure 3b depicts a schematic representation of the methodology employed in this work.

## 3. Electrical Characterization of G-Optrodes Thin Films

**Figure 4:**
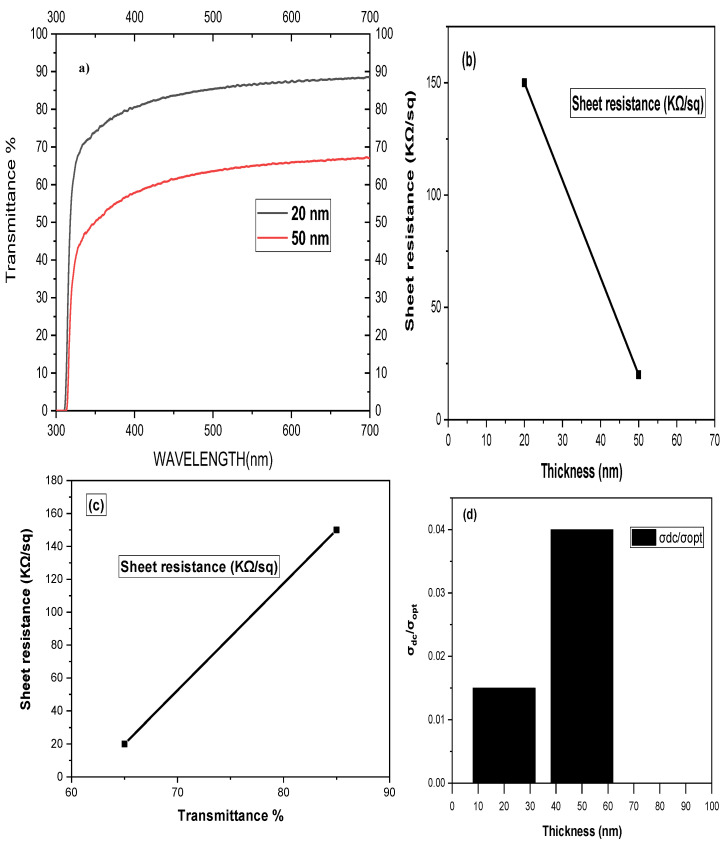
Characteristic plots of G-optrode thin films of different thickness. (**a**) Transmission spectrum, (**b**) sheet resistance vs. thickness plot, and (**c**) plot of sheet resistance vs. transmittance, (**d**) plot of *σ_dc_*/*σ_opt_*.

A UV-Vis-NIR spectrophotometer is used to characterize the above-prepared G-optrodes for transmission in the 300 nm to 700 nm wavelength range of Figure 4a. The transmittance vs. wavelength figure shows that graphene G-optrode sheets are more transparent in the near-infrared range. This is in contrast to poly(3,4-ethylene-dioxythiophene) polystyrene sulfonate PEDOT:PSS and Indium Tin Oxide (ITO) based electrodes, which have lower transmittance towards the Near Infrared (NIR) range [14,43,44]. As a result, graphene becomes more appealing for NIR-based optoelectronic applications. When the thickness of such a film is reduced, the transmittance improves. At different spin rates, G-optrodes were fabricated, yielding thicknesses of 20 nm and 50 nm, respectively. At 550 nm, these films had optical transmittances of 85% and 65%, respectively. The corresponding films were subjected to four-point sheet resistance measurements.

Thinner films are less electrically conductive because there are fewer conduction channels for electron transport. The sheet resistances of the G-optrodes were 150 ± 5 and 20 ± 2 K/sq, corresponding to film thicknesses of 20 nm and 50 nm, respectively, as illustrated in Figure 4b. A plot of sheet resistance vs. transmittance for two different thicknesses is shown in Figure 4c. Sheet resistance and transmittance both rise as film thickness decreases. It is vital to have an excellent optical transmission while maintaining a low sheet resistance when using graphene as transparent electrodes.

This relates to high direct current conductivity (*σ_dc_*) and low optical conductivity (*σ_opt_*) films. The relation between optical transmittance, high direct current conductivity, sheet resistance, and low optical conductivity is given as [45,46]:

(1) $$T={\left[1+\frac{188\;\Omega }{R_{s}}\frac{\sigma_{opt}}{\sigma_{dc}}\right]}^{-2}$$

(2) $$\frac{\sigma_{opt}}{\sigma_{dc}}={\left[\frac{188\;\Omega }{R_{s}}\frac{\sqrt{T}}{\left(1-\sqrt{T}\right)}\right]}^{-2}$$

The preceding equations are used to compute the direct current conductivity to optical conductivity ratio (*σ_dc_*/*σ_opt_*), which is considered a factor of performance for transparent conductors. The calculated values of *σ_dc_*/*σ_opt_* vs. thickness for various films are shown in Figure 4d. G-optrodes thin films of 50 nm have a maximum *σ_dc_*/*σ_opt_* of 0.04, equating to a 65% transmittance and it has a sheet resistance of 20 K/sq.

Except for the film formed at 20 nm, where transmittance increases much more than sheet resistance, the *σ_dc_*/*σ_opt_* value decreases with a reduction in film thickness Figure 4b.

**Figure 5:**
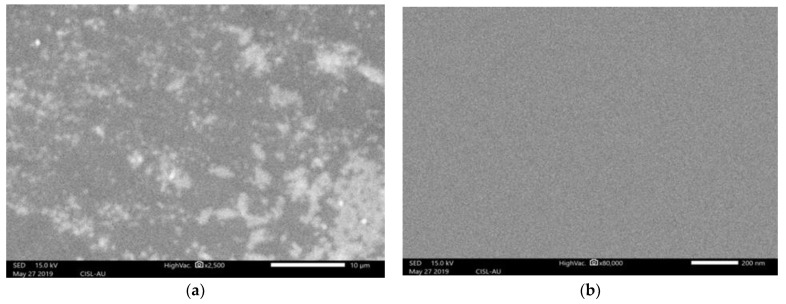
The SEM image of G-optrode thin films at various thickness (**a**) 20 nm and (**b**) 50 nm.

Figure 5a,b depicts the SEM images of the 20 nm and 50 nm thick G-optrodes films, indicating a continuous and homogeneous graphene network. The SEM was used to assess the structural morphology, which revealed homogeneous coverage with fewer wrinkles in G-optrode thin films with thicknesses of 20 nm and 50 nm. At 50 nm layer thickness, compared to 20 nm, the connection between graphene and glass looks to be pretty excellent.

### Stability Evaluation of G-Optrode Thin Film’s

**Figure 6:**
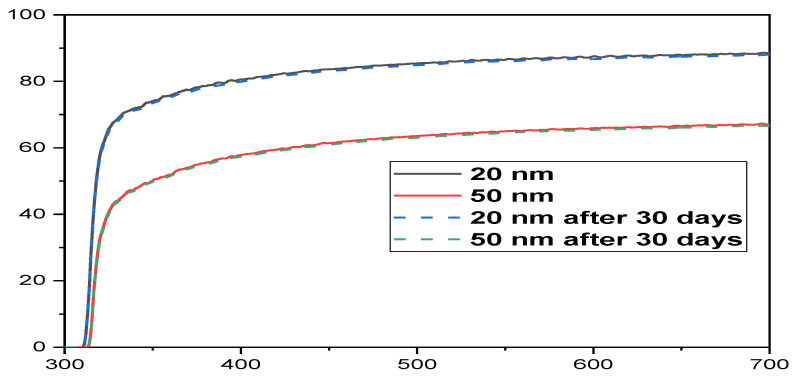
G-optrode transmission (on *y*-axis) spectrum with thicknesses of 20 and 50 nm (). Straight lines (__) represent the G-optrode sheet before PBS immersion, while dotted lines (---) represent after 30 days of PBS immersion from 300 to 700 nm wavelength range (on *x*-axis).

The optical absorbance of the electrodes was evaluated both directly after fabrication (day 0) and after one month to determine whether the graphene layer’s optoelectronic properties were maintained throughout processing and preservation in a saline condition (day 30). In PBS, the samples were immersed and exposed to environmental light and dark cycles for 12 h at 37 °C. Figure 6 depicts the transmission spectrum of fabricated samples with thicknesses of 20 nm and 50 nm before and after 30 days of PBS immersion, which was nearly identical to the absorption. The optical absorption spectrum also showed no significant changes. The stability of the graphene coating with 20 nm and 50 nm thicknesses over the glass was good after 30 days of immersion in PBS solution; the morphology of the graphene layer coating on both thin films remains unchanged, and its electrical and optical properties are fully preserved.

**Figure 7:**
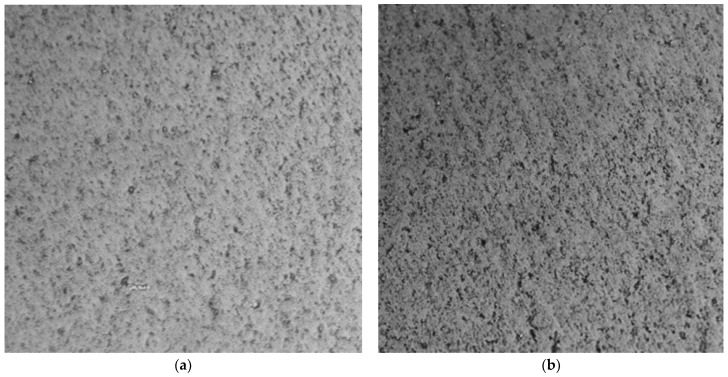
Microscopic image (Nikon TS 100 Eclipse) of the G-optrodes with (**a**) 20 nm and (**b**) 50 nm, respectively after 30 days of PBS immersion.

The G-optrode film microscopic image is shown in Figure 7, which clearly reveals that there is no change in morphology and that it has the same electrical and optical properties before immersion in PBS.

## 4. Optical Stimulation via G-Optrode Substrate Bio Interfaces: Potential Mechanisms and Analysis

G-optrode films with 20 nm and 50 nm thicknesses were used without additional purification. The capacitive or charge distribution due to temperature gradients caused by local heating, the Faradic effect, can all cause optical stimulation. The goal of this research is to determine whether optical stimulation through bio-interfaces is influenced by capacitive (charge redistribution) or Faradaic (charge transfer occurs in the electrolyte solution as a result of chemical reactions and photo-generated electrons) mechanisms. The pH of the fluid changes once Faradaic charge transfer takes place in the G-optrode electrolyte [12].

**Figure 8:**
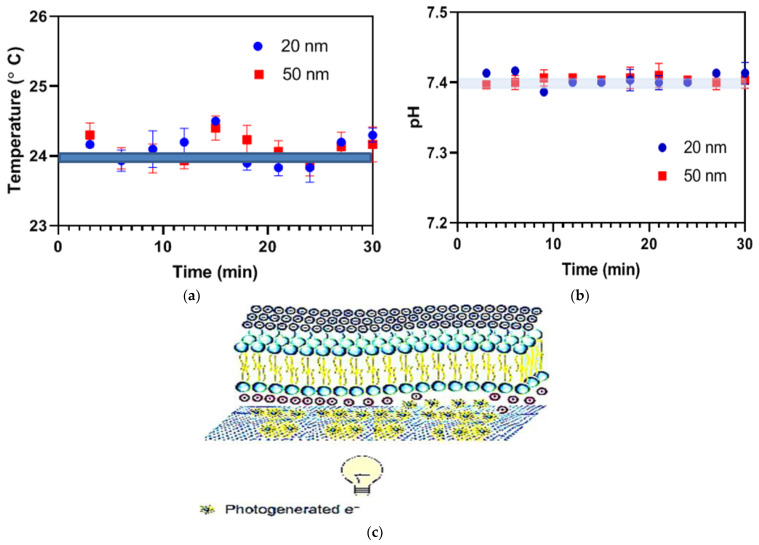
Testing for the existence of the faradic effect via G-optrodes (**a**) surface temperature of G-optrodes, (**b**) pH of an electrolytic solution covering the G-optrodes, and (**c**) schematic illustrating the proposed mechanism using G-optrodes bio-interfaces.

A Faradaic reaction can cause unwanted severe cell degradation and electrode damage as a result of a redox electrochemical reaction involving living organisms in solution and electrodes. As a result, non-faradic charge transfer photo-stimulation is always preferable. A pH reading is the most accurate way of determining this. The pH of the electrolytic solution containing immersed G-optrodes remained constant after 30 min of continuous light illumination, as evaluated directly after fabrication (day 0) and one month later (day 30). Figure 8b strongly indicates that the Faradaic mechanism is not at work [12,47]. This is due to the fact that the authors did not use a voltage bias, and photogenerated electrons in the graphene layer have a lifetime in the femtosecond range. It is expected that photogenerated currents will be negligible [48,49].

Temperature gradients caused by the local heating result in charge transfer is another possible scenario of optical stimulation [50]. The researchers measured the surface temperature of G-optrodes after 30 min of continuous light exposure [51,52] to see whether photo-stimulation is mediated by a temperature process and found no differences in temperature. For the reasons stated below, direct light-induced or heat-mediated consequences are extremely improbable in this setting: Light-induced heat tends to produce a progressive changeable cellular response throughout continuous exposure to light [53]; meanwhile, the authors observed a rapidly attained stable response.

Figure 8a shows that the surface temperature of G-optrodes did not change after a continuous 30 min exposure to light (n = 3), recorded immediately after fabrication (day 0) and after one month (day 30) with a 100 mW/cm*^2^* illuminance. The data will be presented as mean SEM. Figure 8b demonstrates that there were no changes in the pH of an electrolytic solution covering the G-optrode with a thickness of 20 nm and 50 nm surface throughout a continuous 30 min exposure to light (n = 3). Authors have examined the changes immediately after fabrication (day 0) and after one month (day 30) with a 100 mW/cm*^2^* irradiance. The schematic depicting the proposed methodology of optical stimulation of cells employing light-activated G-optrode bio-interfaces is shown in Figure 8c.

The next scenario is that the optical stimulation is solely based on capacitive mechanisms. This process is characterized by the formation of Helmholtz layers with opposing charges at the electrolyte/G-optrode and cell/electrolyte interfaces. There is no charge transfer between the electrolyte and the G-optrode in this process. Capacitive stimulation is distinguished during biocompatibility studies by the absence of any detrimental consequences, such as the deterioration of the G-optrode film or cell cultures. As a result, capacitive coupling holds the stimulation function. These findings show that G-optrode thin films can be used to stimulate neuronal activity [54].

### Evaluation of G-Optrode Thin Film Bio-Organic Interface Behaviour

**Figure 9:**
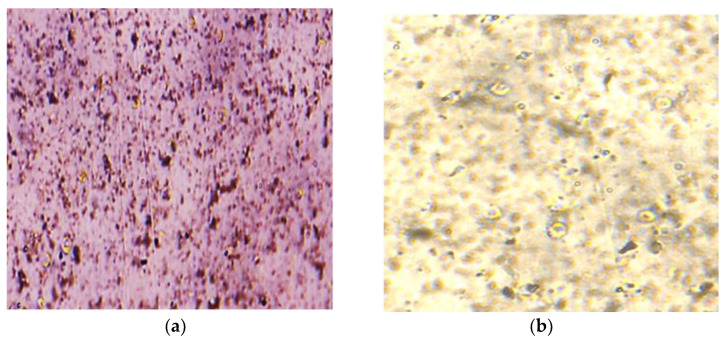
PC-12 cells were seeded on 20 nm thick G-optrode samples. (**a**) Prior and (**b**) following MTT treatment.

**Figure 10:**
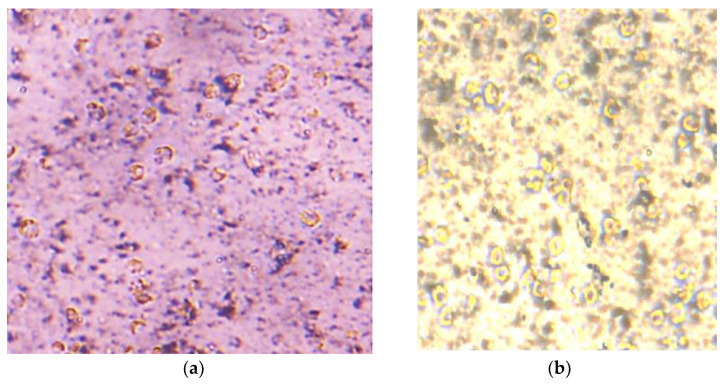
PC-12 cells were seeded on 50 nm thick G-optrode samples. (**a**) Prior and (**b**) following MTT treatment.

**Figure 11:**
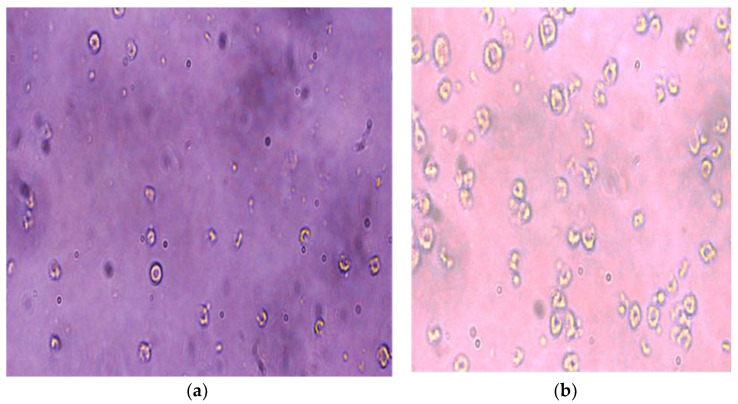
PC-12 cells were seeded on control glass substrate. (**a**) Prior and (**b**) following MTT treatment.

**Figure 12:**
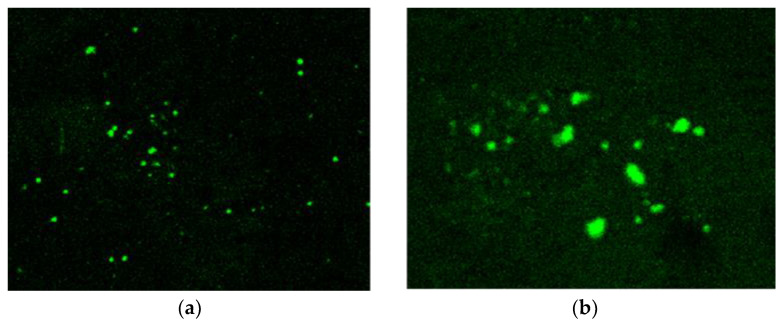
Confocal microscopic image of PC-12 cells grown on a G-optrode (**a**) sample and (**b**) stained with AO (green).

The thickness of the graphene coating over a glass substrate is 20 nm and 50 nm, and the transmittance of the G-optrode substrate is 80% and 65%, respectively, and it was used without any further purification. In this work, the authors utilized G-optrode thin film, which shows great potential, such as stability, uniformity, and transparency. In order to determine the biocompatibility of G-optrodes of various thicknesses. PC-12 cells were grown for up to 7 days on glass substrates with the graphene coating and on control glass substrates Figure 9, Figure 10 and Figure 11 the images were taken using a Nikon Ts eclipse microscope with a magnification of ×20. As described in Section 2, G-optrodes with 20 nm and 50 nm thicknesses were prepared, as well as control glass samples. In cell culture flasks, rat adrenal pheochromocytoma (PC-12) cell lines were grown. When PC-12 cells reached confluence, they were enzymatically disseminated utilizing trypsin–EDTA and plated on G-optrodes substrates of varying thicknesses at a volume of 15,000 cells/well. The adherence and pattern growth of neuronal cells upon this control glass surface and G-optrodes were almost equivalent under all circumstances. The researchers did not utilize any adhesion proteins in this experiment since PC-12 cells are inherently adhesive to the substrate and the seeded cells adhered well to the surface throughout all circumstances. However, adhesion enzymes have been used by researchers in several of the previously reported works [54]. The MTT (thiazolyl blue tetrazolium bromide) test and Acridine Orange (AO) stains were conducted at various times in culture to evaluate cell viability over 20 nm and 50 nm G-optrode substrates in comparison to control substrates. An MTT test with three replicates has been used to analyze the proliferation of grown PC-12 cells on various substrates on days 1, 4, and 7. The viability of feasible cells was determined by staining them with AO. The confocal image of PC-12 cells grown on a G-optrode sample and stained with AO (green) is shown in Figure 12.

The culture mediums were changed with a new medium that did not include FBS or phenol red, as well as an MTT test at a concentration of 0.5 mg/ml. The cells were again incubated overnight at 37 °C. The capacity of cells cultured in culture well plates to change soluble MTT into an undissolved purple formazan reaction product has been used to measure cell viability [55,56].

Figure 9, Figure 10, Figure 11 and Figure 12 test the biocompatibility of G-optrodes. Representative light microscopy images of rat pheochromocytoma before MTT assay treatment (left) and after MTT assay treatment (right) cultured on a graphene-based G-optrodes substrate with 20 nm, 50 nm, and control.

Cells were uniformly seeded onto G-optrode substrates of 20 nm and 50 nm, as well as control wells of a flat-bottomed plate with growth medium, and incubated at 37 °C in a humidified 5% CO_2_ environment. The cells were rinsed with PBS after the medium was removed and incubated with MTT overnight at 37 °C in an incubator humidified with 5% CO_2_ and 95% air. After removing the MTT solution, dimethyl sulfoxide (DMSO) was used to dissolve the water-insoluble formazan. A multimode reader was used to measure the MTT absorptions at 550 nm. The MTT conversion into purple formazan was used to determine the relative percentages of viable cells (compared to non-treated controls).

**Figure 13:**
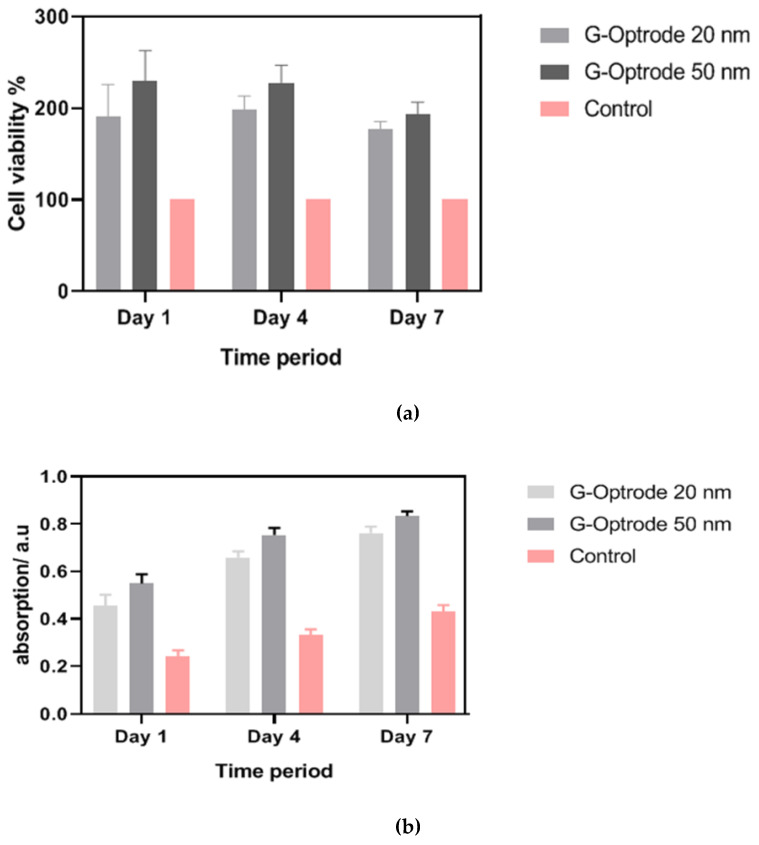
Cell viability on G-optrodes. (**a**) Absorbance and (**b**) percentage.

Figure 9, Figure 10 and Figure 11 show images of cells cultured on various substrates prior to and following MTT treatment. The obtained results reveal that there have been no significant differences in absorbance and cell viability on 20 nm and 50 nm G-optrode substrates compared to control glass substrates (Figure 13b, one-way Analysis Of Variance (ANOVA), n = 3, *p* = 0.0080) and absorbance under all culture conditions (Figure 13a, ANOVA, n = 3, *p* = 0.923), indicating that the bio-interface involves changing neither neuronal network development nor the neuronal viability even over a significantly long duration.

The absorption of formazan up to 7 Days in Vitro (DIV) is depicted in Figure 13a at three different time points. MTT can be converted into formazan by mitochondrial hydrogenases in living cells, which have a high absorption value. In all cases, increasing the incubation time tends to increase cell proliferation and absorbance after seven days, as predicted. The obtained result clearly illustrates that cell proliferation in 20 nm and 50 nm G-optrode substrates was as high as in the control glass after 7 DIV; it also confirms that the G-optrode bio-interfaces used in this work for cell culture have very low cytotoxicity. Figure 13b compares the percentage of cell viability at each time point for 20 nm and 50 nm G-optrode substrates and glass control substrates.

After 7 DIV, cell growth in G-optrode substrates with thicknesses of 20 nm and 50 nm was as high as in the glass positive control, validating the low cytotoxicity of these graphenes as cell culture substrates. As complementary information, Figure 13b depicts the percentage vitality of cells in comparison to the equivalent glass control substrates at each time point.

After the above process, it has been realized that the cell density is significantly higher on G-optrode substrates with the thickness of 20 nm and 50 nm than on control substrates, moreover, the cell density in 50 nm G-optrode substrate is higher than 20 nm G-optrode substrate, whereas the cell viability and contractile profiles slight differences 50 nm is superior to the 20 nm. This study’s excellent biocompatibility of G-optrode based bio-interfaces is in agreement with previous research works.

## 5. Conclusions and Future Recommendation

This research utilizes a spin coating technique to fabricate G-optrode thin films. The fabricated G-optrodes film exhibited homogeneous, extremely thin films with good transparency. The electrochemical characterization of the fabricated films shows that the G-optrode thin films have a high specific capacitance and might be highly useful in a wide variety of applications, including supercapacitors.

This research examines the prospect of extending the G-optrodes bio-interface. Two G-optrodes with 20 nm and 50 nm thicknesses have been realized as candidates for optically stimulating living cell electrodes in a biological environment using thin film electrodes. The authors characterized the electrochemical stability, surface morphology, biocompatibility, interface capacitance, and lastly, the unique photoexcitation functionality in this analysis. The G-optrodes demonstrated good qualities such as stability, biocompatibility, and cell photoexcitation capabilities. The G-optrodes with thicknesses of 20 nm and 50 nm were both capable of optically modulating the membrane potential in accordance with the previously reported thermal effect. Both G-optrodes were able to modify the membrane potential optically as per the thermal effect, as previously reported work using different materials under similar conditions.

This work demonstrates that G-optrodes optical stimulation thin film electrode could enable thermal sterilizing methods, implying that successful cell culturing and proliferation may be accomplished simply. Overall, this work provides the necessary foundation for the development of G-optrodes optical stimulation thin film electrode-based interfaces for optical modulation of cellular electrical activity across the visible spectral window.

Though this research has brought in valuable advancements in optical stimulation using thin film electrodes research, it is not without shortcomings. Further studies on animals will be essential to further ascertain that G-optrode optical stimulation technology is indeed a reliable alternative for bio-interface applications.

The future G-optrode devices should be realized in soft/flexible substrates. For in vivo tests in the future, especially long-term implants, significant improvement in the device will be required, such as realizing the device in flexible/flat substrate, optimization, encapsulation, and in vivo biocompatibility tests.

These studies have been determined by a variety of fabrication, electrical characterization, and biological study experiments. Despite the fact that the experimental findings suggest a possible concept for the next generation of optical stimulation bio-interfaces, there are limitations that need to be addressed, which will be the focus of future work. The future device may provide evidence towards the idea of developing successful bio implant electrodes, which are more realistic and applicable by making these improvements.

## Data Availability

All data and material used to prepare this manuscript are available in this document.
